# Etymologia*: Orientia tsutsugamushi*

**DOI:** 10.3201/eid1202.ET1202

**Published:** 2006-02

**Keywords:** Etymology, Etymologia, Orientia tsutsugamushi

## [or´´e-en´she-ə (t)süt´´sə-gə-mü´she]

Etiologic agent of scrub typhus, transmitted by the bite of thrombiculid mite larvae. From the Latin *orien*s, "east" and the Japanese *tsutsuga*, "sickness" plus *mushi*, "insect." The disease was first documented in China in 313 AD and has been a frequent cause of illness in soldiers stationed in the western Pacific. In Vietnam, *O. tsutsugamushi* was among the most common causes of fever in soldiers.

**Sources:** Dorland's illustrated medical dictionary. 30th ed. Philadelphia: Saunders; 2003; Merriam-Webster's collegiate dictionary. 11th ed. Springfield (MA): Merriam-Webster Incorporated; 2003; and Raoult D. Scrub typhus. In: Mandel GL, Bennett JE, Dolin R, editors. Principles & Practice of Infectious Diseases. 6th ed. Churchill Livingstone; 2004. p. 2309-10.

